# The association between implementation of multidisciplinary rounds and clinical outcomes

**DOI:** 10.3389/fcvm.2022.1005150

**Published:** 2022-11-04

**Authors:** Pranati Sreepathy, Yoo Jin Kim, Zaneta Ahuja, Adhir R. Shroff, Noreen T. Nazir

**Affiliations:** ^1^Department of Medicine, University of Illinois at Chicago, Chicago, IL, United States; ^2^The Johns Hopkins Hospital, Johns Hopkins Medicine, Baltimore, MD, United States; ^3^College of Medicine, University of Illinois at Chicago, Chicago, IL, United States; ^4^Health Sciences System, University of Illinois Hospital, Chicago, IL, United States; ^5^Division of Cardiology, Department of Medicine, University of Illinois at Chicago, Chicago, IL, United States

**Keywords:** multidisciplinary team, mortality benefit, cardiovascular care and outcomes, care strategies, cardiovascular care coordination

## Abstract

**Background:**

Multidisciplinary rounds (MDR) consisting of social workers, dietitians, pharmacists, physical therapists, nurses, and physicians have been implemented at many healthcare institutions to address the complex components of inpatient care. However, little is known on the association of MDR on clinical outcomes across cardiovascular pathologies. This study aimed to investigate the impact of MDR on cardiovascular patients.

**Methods:**

Hospital admissions to inpatient cardiology were evaluated prior to (November 2017 to November 2018) and after implementation of MDR (December 2018 to August 2020) at a metropolitan academic medical center. The following outcomes were evaluated: clinical complications (incidence of stroke, gastrointestinal bleed, myocardial infarction, or systemic infection during hospitalization), Length of Stay (LOS), 30-day readmissions and all-cause in-hospital mortality. Secondary outcomes included utilization of physical therapy and dietary services.

**Results:**

Admissions were evaluated prior to (*N* = 1054) and after (*N* = 1659) MDR implementation. All-cause in-hospital mortality after MDR implementation decreased significantly from 2.8 to 1.6% (*P* = 0.03). Although the number of complications and LOS decreased, these differences were not statistically significant. No significant change was observed in 30-day readmissions. Significant increase in the utilization of physical therapy (34.2 to 53.5%; *P* < 0.01) and dietary services (7.2 to 19.3%; *P* < 0.01) were observed.

**Conclusion:**

Multidisciplinary rounds implementation was associated with significantly decreased mortality and positively impacted resource utilization with increased consultations for ancillary services. MDR is a high impact intervention that utilizes existing resources to improve mortality and should be implemented especially for cardiovascular patients. Further investigation into the benefit of MDR across different patient populations and care settings is warranted.

## Introduction

Patients with cardiovascular disease who are hospitalized often have a high burden of comorbidities that require engagement with multiple disciplines in the healthcare system. The complexities of cardiovascular disease management in the acute setting is evidenced by increased hospital readmissions and in-hospital mortality (1). A driving factor in hospital readmissions and mortality among patients hospitalized due to cardiovascular disease is the high burden of comorbidities that require engagement with the healthcare system in many different disciplines (1). As the burden of cardiovascular disease continues to increase with an aging population, hospital care for the cardiac patient will become more complex (2, 3). These patients benefit from a multifaceted approach to treatment components separate from clinical care such as physical activity, exercise, diet and post-hospital follow up being contributory to overall hospital stay (4). Thus, a vast majority of hospitalized patients have significant care gaps given the multidisciplinary care needs (5, 6). For example, extended periods without nutrition or delayed attempts at patient mobilization can lead to a decrease in functional status, subsequent deconditioning and prolonged hospitalizations (7, 8).

Healthcare systems have started to implement Multidisciplinary Rounds (MDR), which have been shown to improve quality metrics and decrease operating costs (9). MDR often consist of nurses, social workers, discharge planners, physical therapists, dietitians, pharmacists, physicians and/or physician extenders who systematically address the daily care plan, discharge needs, and care gaps of the hospitalized patient. MDR improve communication among healthcare members leading to increased coordination of care and decreased hospital costs (10). Use of checklists to incorporate topics of focus have been studied as an effective way to guide MDR and engage members of the patient care team (11).

Currently, little research exists to understand the impact of MDR implementation on clinical outcomes such as clinical complications, length of stay (LOS), 30-day readmissions, and all-cause in-hospital mortality. Additionally, the impact on resource utilization from ancillary services has not been evaluated. Furthermore, prior research regarding MDR has been limited to only patients with heart failure with reduced ejection fraction (HFrEF) and ischemic heart disease (12). In this study, we sought to understand the association of MDR implementation on the clinical outcomes in the cardiac population in a metropolitan academic hospital.

## Methods

Clinical data encompassing inpatient hospital encounters of patients admitted to the cardiology inpatient service at the University of Illinois at Hospital and Health Sciences System (UI Health) between November 2017 and August 2020 was evaluated. The study period was divided into two time periods, Before MDR Implementation (November 2017 to November 2018) and After MDR Implementation (December 2018 to August 2020). All adult patients admitted to cardiology inpatient service and all levels of care such as medical-surgical floor and intensive care unit (ICU) were included. Patients under Hospice Care and under the age of 16 were excluded. Throughout the study period, input from the care team was gathered and implemented into MDR. Physical therapists and nutrition services (clinician dieticians) were included in MDR in December 2018.

### Multidisciplinary team and rounds

The multidisciplinary team consisted of clinical providers (resident, fellow, and/or attending physicians), pharmacists, physical therapists, social workers, nurses, dietitians, and care coordinators (focusing on discharge planning and follow up appointments). Patients were presented by the clinical provider and all participants were invited to share input regarding patient care and resource utilization using a standardized checklist (Supplementary Figure 1). MDR checklist and process was derived from the American Heart Association’s Get With the Guidelines (AHA GWTG) programs in heart failure and coronary artery disease as the implementation of the programs have been demonstrated to improve mortality (12). Medication adherence, patient education and post-hospitalization care were universally addressed and emphasized for patients regardless of specific cardiovascular diagnosis. MDR also aimed to improve institutional adherence to the American Heart Association’s initiative.

### Outcomes

The primary outcomes of this study were clinical complications (stroke, gastrointestinal bleed, myocardial infarction, or systemic infection during hospital stay), LOS, 30-day readmissions and all-cause in-hospital mortality. Secondary outcomes included number of ancillary staff consults (dietary services, physical therapy) after MDR implementation.

### Statistical analysis

Mean (standard deviation) and proportions were utilized to describe the study population and clinical outcomes. Independent sample *t*-tests and chi-square tests were used as appropriate to compare clinical outcomes between admissions that occurred before and after the implementation of MDR. Normality of data was assessed by histogram as bell-shaped distribution was observed. Two-sided *P*-value of < 0.05 was considered statistically significant. Analyses were completed using R studio (Version 1.4.1717, RStudio), and figures were developed with Plotly (2015, Plotly Technologies).

## Results

A total of 2,713 hospital admissions occurred during the study period of which 1,051 and 1,659 admissions occurred before and after the implementation of MDR, respectively. Among all patients who were admitted during the study period, the mean age was 62.5 years. Of this population 49.6% were female and 57.5% Non-Hispanic Black (Table 1). Majority of the study population were either on Medicare, Medicaid or uninsured (50.4%). The incidence of women higher in the population before MDR implementation, however, there were no statistical differences in baseline characteristics such as comorbidities, primary admitting diagnosis, race, age, or insurance status (Table 1).

**TABLE 1:** Characteristics of patients prior to and following the implementation of multidisciplinary rounds.

	All (*N* = 2710)	Before MDR (*N* = 1051)	After MDR (*N* = 1659)	*P-*value
Age, mean ± SD, years	62.5 ± 15.1	62.4 ± 15.2	62.7 ± 15.0	0.67
Female, no. (%)	1,345 (49.5)	549 (52.2)	793 (47.8)	0.03
Race, no. (%)				0.23
Black	1,557 (57.5)	622 (59.2)	935 (56.4)	
White	307 (11.3)	123 (11.7)	184 (11.1)	
Asian	45 (1.7)	19 (1.8)	26 (1.6)	
Other	801 (29.6)	287 (27.3)	514 (31.0)	
Insurance status				0.05
Medicare/Medicaid	1,271 (46.9)	523 (49.8)	748 (45.2)	
Other	1,345 (49.6)	496 (47.2)	849 (51.2)	
Uninsured	94 (3.5)	32 (3.0)	62 (3.7)	
Primary diagnosis				
Atrial fibrillation/flutter	356 (13.1)	143 (13.6)	213 (12.8)	0.60
Angina	103 (3.8)	51 (4.9)	52 (3.1)	0.74
Congestive Heart Failure^1^	715 (26.4)	267 (25.4)	448 (27.0)	0.29
NSTEMI^2^	65 (2.4)	23 (2.2)	42 (2.5)	0.99
STEMI^3^	61 (2.3)	14 (1.3)	47 (2.8)	0.51
Comorbidities				
Chronic kidney disease	1,072 (39.6)	408 (38.8)	664 (40.0)	0.56
Hypertension	1,060 (39.1)	430 (40.9)	630 (38.0)	0.14
Dyslipidemia	1,438 (53.1)	561 (53.4)	877 (52.9)	0.82
PCI^4^	99 (3.7)	39 (3.7)	60 (3.6)	0.98
TAVR^5^	9 (0.3)	4 (0.4)	5 (0.3)	0.99
Atrial fibrillation	931 (34.4)	365 (34.7)	566 (34.12)	0.78
Diabetes Mellitus	1,405 (51.9)	538 (51.2)	867 (56.3)	0.61

^1^Including systolic, diastolic, and unspecified.

^2^Non-ST elevation myocardial infarction.

^3^ST elevation myocardial infarction.

^4^Percutaneous coronary intervention.

^5^Transcatheter aortic valve replacement.

Multidisciplinary rounds implementation was initiated in December 2018. The proportion of hospital admissions associated with clinical complications (3.9 to 2.9%; *P* = 0.19) decreased after the implantation of MDR although the difference was not statistically significant. Furthermore, there was not a significant difference in the average LOS when comparing LOS before and after the implementation of MDR (4.2 vs. 4.4 days; *P* = 0.21). There was also no significant difference in the proportion of admissions associated with a readmission within 30 days before and after the implementation of MDR (7.7 vs. 8.7%; *P* = 0.37). All-cause mortality following the implementation of MDR significantly decreased (2.8 to 1.6%; *P* < 0.03). Additionally, the proportion of admissions associated with the placement of an ancillary consult to physical therapy (34.2 to 53.5%; *P* < 0.01) and nutrition services (7.2 to 19.3%; *P* < 0.01) significantly increased (Table 2).

**TABLE 2:** Clinical outcomes before and after the implementation of MDR.

	Before MDR *N* = 1054	After MDR *N* = 1659	*P*-value
Length of stay, mean ± SD, days	4.2 ± 4.5	4.4 ± 4.4	0.21
Cases with complications, no. (%)	41 (3.9)	48 (2.9)	0.19
30-day readmissions, no. (%)	81 (7.7)	145 (8.7)	0.37
Mortality, no. (%)	30 (2.8)	26 (1.6)	0.03
Length of stay, mean ± SD, days	4.2 ± 4.5	4.4 ± 4.4	0.21
Physical and Occupational Therapy, n/N (%)	79/231 (34.2)	552/1036 (53.3)	<0.01
Nutrition, n/N (%)	111/1545 (7.2)	100/517 (19.3)	<0.01

## Discussion

Previously there has been little research demonstrating the impact of MDR implementation on mortality. Prior studies evaluating MDR implementation were limited to patients hospitalized for ischemic cardiomyopathy and ACS. In this evaluation, the impact of MDR on admissions related to a broad range of cardiac etiologies including HFpEF, MI (NSTEMI, STEMI), cardiac dysrhythmias and unstable angina. The implementation of MDR in an inpatient cardiology service resulted in significantly decreased all-cause mortality while increasing ancillary staff utilization.

We found that 30-day all cause readmissions did not significantly decrease with the implementation of MDR (7.7 vs. 8.7%; *P* = 0.37). Decreased 30 day readmissions in response to MDR has only been demonstrated in a single center retrospective study evaluating HFrEF due to ischemic heart disease patients, but no change in HFpEF or non-ischemic cardiomyopathy after the implementation of MDR (12, 13). This is consistent with the results seen in our study given total 30 day all cause readmission across all cardiac patients admitted to the service line did not change with MDR implementation. These results may be explained by a couple factors. There are extensive quality metrics focusing on follow up and readmissions for HFrEF in comparison to other disease processes such as non-ischemic cardiomyopathy and HFpEF (6) and less data for these interventions in other pathologies such as HFpEF, non-ischemic cardiomyopathy, refractive atrial fibrillation, and microvascular disease and angina.

Though we did not observe a statistically significant decrease in the LOS following the implementation of MDR, there was a significant decrease in mortality and the rate of clinical complications. MDR bring attention to treatment gaps that may prolong LOS and provide an avenue for multiple healthcare providers to discuss patient care. This collaboration can lead to the early prevention of adverse events and complications as the proportion of all-cause deaths and clinical complications decreased following the implementation of MDR. Furthermore, these findings are consistent with previous studies that did not demonstrate a significant decrease in average LOS following the implementation of MDR among patients admitted with diagnoses of acute coronary syndrome or heart failure (7, 13).

**FIGURE 1:**
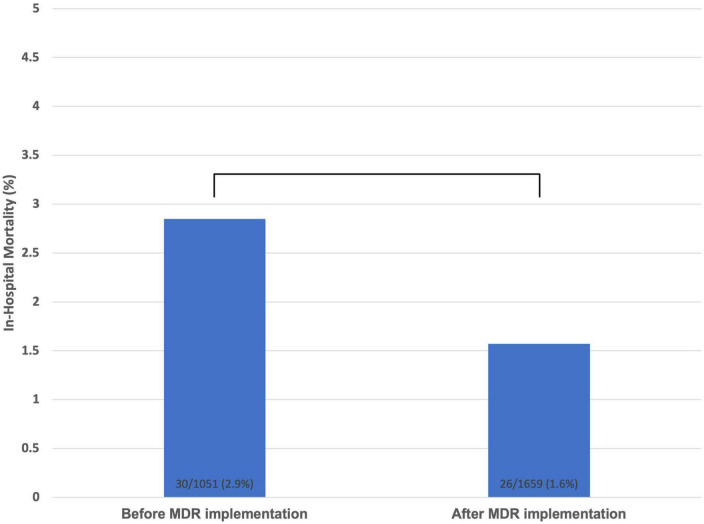
All-cause in-hospital mortality prior to and after multidisciplinary rounds implementation.

**FIGURE 2:**
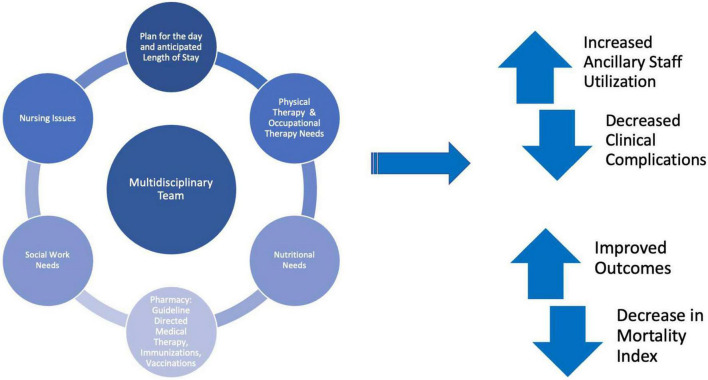
Multidisciplinary rounds components and benefits.

Multidisciplinary rounds implementation resulted in significantly decreased all-cause mortality (2.8 to 1.6%; *P* < 0.03) (Figure 1). To our knowledge there is currently no data on the impact of MDR on mortality for patients hospitalized with cardiovascular disease other than heart failure (12, 13). Cardiovascular disease continues to be a leading cause of global mortality and there is urgent need for effective and fiscally sound care strategies (14). This study further cements MDR as one of these initiatives as many hospital systems have many of the components of MDR already in place such as social work, dietary, physical therapy, and pharmacy (Figure 2).

Physical therapy interventions have been demonstrated to reduce all-cause hospital readmissions and mortality (3, 15). Early mobilization in the inpatient setting has been shown to improve clinical outcomes and improve patients’ functional status at discharge (16). Furthermore, physical therapy and cardiac rehabilitation have been shown to be associated with risk reduction of all-cause mortality after myocardial infarction regardless of socioeconomic status, sex, age or comorbidities. (17). We found that the number of consults to physical therapy and in turn the referrals to cardiac rehabilitation increased from 34.2 to 53.5%; *P* < 0.01.

Increased dietary counseling has been shown to have benefits in reducing cardiovascular risk (18). Dietary counseling has also been found to be beneficial among heart failure patients and increase adherence with a sodium restricted diet (19). The implementation of MDR increased the number of consults to nutrition services from 7.2 to 19.3%; *P* < 0.01. By incorporating these services into MDR, appropriate care and counseling is ensured for patients along with providing a contact for further information for these services.

A recent quantitative systemic review of multidisciplinary rounding in acute care settings found that majority of the studies included a statistically significant increase in staff satisfaction after implementation of MDR specifically among house staff and nursing (7, 20). The effect of MDR on increased collaboration between members of the clinical care teams is also seen in this study. With dedicated opportunity for participation and clear communication between care members, the team is more likely to better utilize services they can provide to better care for the hospitalized patient. MDRs facilitate utilization of available ancillary services for patient care and are an avenue for discussion of patient care by all involved in hospital care. Being able to organize these services into the process outlined in this study engages several care providers, allowing an opportunity for discussion to positivity impact patient care and hospital experience. Future studies evaluating how MDR may impact qualitative measures such as patient experience during the hospital admission will hopefully continue to show impact on these metrics.

### Study limitations

This study was conducted at a single academic medical center that reduces external validity. Evaluation of MDR implementation was completed retrospectively with a lack of randomization to the intervention. Additionally, we utilized only the electronic health record of the study site that may have limited our ability to evaluate the rate of readmission. Furthermore, the institution changed the electronic medical record system during the study period (September 2020) that may have led to fragmented timeline for certain parameters.

### Clinical perspective

This study demonstrates the effectiveness of comprehensive clinical care and ancillary staff utilization. We found that the implementation of a multidisciplinary clinical team enhances quality patient care and addresses treatment gaps to ultimately lead to a decrease in all-cause mortality among cardiac patients. Our findings demonstrate the significant impact of MDR implementation on hospital care.

## Conclusion

The implementation of MDR facilitated a statistically significant decrease in overall all-cause mortality and increase in ancillary staff utilization in the care of the hospitalized cardiac patient. Engaging care providers in a systematic process allows for care providers to provide collaborative, patient centered care and is effective in impacting clinical care outcomes with decreased complications and improved patient survival.

## Data availability statement

The raw data supporting the conclusions of this article will be made available by the authors, without undue reservation.

## Author contributions

PS and NN: study conception and design. ZA: data collection. YK: analysis. PS, YK, AS, and NN: draft manuscript and preparation. All authors reviewed the results and approved the final version of the manuscript.
