# Supplementary figures and images for: The association between implementation of multidisciplinary rounds and clinical outcomes
Source: Front Cardiovasc Med. 2022 Nov 4;9:1005150. doi: 10.3389/fcvm.2022.1005150 (PMC9671934; doi:10.3389/fcvm.2022.1005150)

**SUPPLEMENTAL**


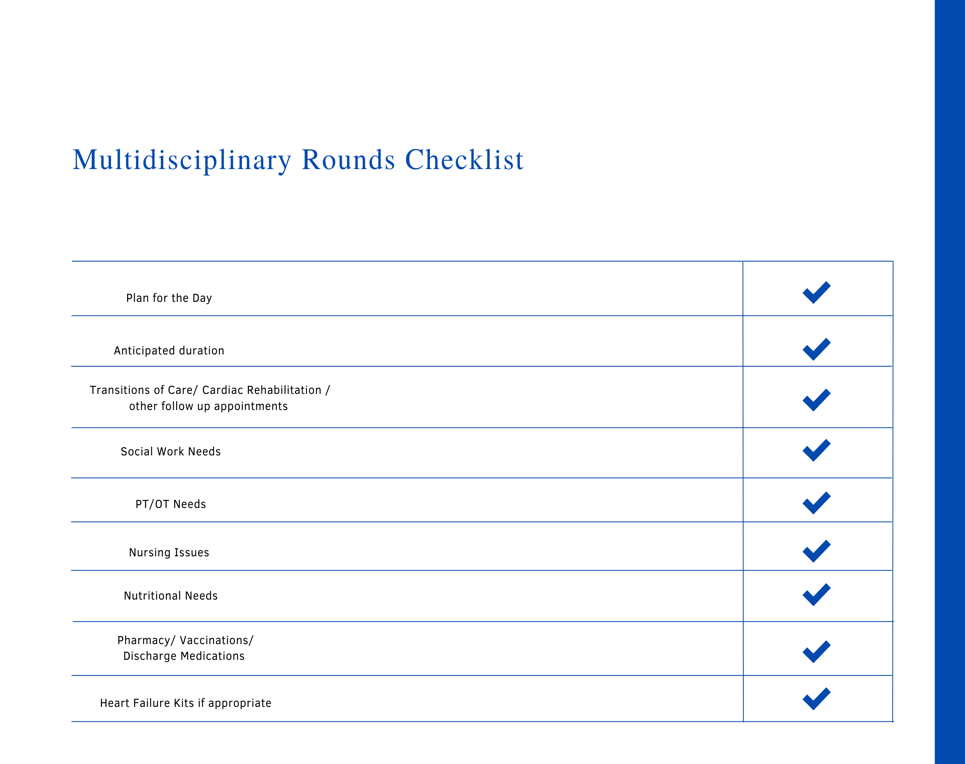


Supplemental Figure 1: MDR checklist used daily during inpatient rounds

Supplement: Supplementary file 1 [file Table_1.DOCX]
